# HIV-associated sensory neuropathy: current progress and future challenges

**DOI:** 10.1515/nipt-2025-0010

**Published:** 2025-12-11

**Authors:** Isaac J. Gamez, Ajay Pal, Connor Haines, Subo Yuan

**Affiliations:** John Sealy School of Medicine, University of Texas Medical Branch, Galveston, TX, USA; Department of Neurobiology, University of Texas Medical Branch, Galveston, TX, USA; Institute for Human Infections and Immunity (IHII), University of Texas Medical Branch, Galveston, TX, 77555, USA

**Keywords:** HIV, pain, pathways, neuropathy, HIV-associated sensory neuropathy, antiretroviral therapy

## Abstract

Currently, no US Food and Drug Administration-approved treatments exist to manage HIV-associated sensory neuropathy (HIV-SN) and management is largely confined to adjusting antiretroviral therapy (ART) doses and medications. Thus, this urgent health crisis requires strong research commitment to identify a cure or palliative treatment. This review explores the current state-of-the-art related to HIV-SN. It first explores recent developments in the understanding of HIV-SN, emphasizing the importance of developments in the HIV-SN mouse model and non-myelination intra epidermal never fiber denervation. Next, the neurotoxic side effects of ART are summarized. Finally, we explore the interactions and synergy between HIV-SN and ART in the pathogenesis of peripheral neuropathy. While the overall mortality related to HIV has decreased significantly in recent decades, further elucidation of the exact mechanisms of HIV-SN is needed to better treat patients living with HIV as a chronic condition.

## Introduction

By the end of 2023, the Joint United Nations Programme on HIV/AIDS *and the World Health Organization* reported that an estimated 39.9 million people worldwide live with HIV [1]. Global access to antiretroviral therapy (ART) has turned HIV infection from a deadly disease into a controllable, chronic disease and closed the gap in lifespan between people living with HIV infection (PLWH) and the general population [2]. Unfortunately, the prevalence of HIV associated pain (HIV-PAIN) does not dramatically decrease when ART has effectively suppressed the HIV viral viremia [3]. The presence of under-addressed HIV-PAIN, including HIV associated sensory neuropathy (HIV-SN), is demoralizing, dulls quality of life and, importantly, disrupts ART adherence [4].

The difficulties of chronic HIV infection are not only related to immune deficiency but also to neurological sequelae such as peripheral neuropathy, cerebrovascular disease, and neurocognitive impairment [5], [6], [7]. In the progression of HIV infection, there is an increased risk of HIV-SN due to residual viral mediated neuroinflammation [8], [9], [10], [11], [12]. HIV-SN ranges in clinical presentation from numbness and tingling all the way to debilitating neuropathic pain that can interfere with activities of daily living, resulting in social consequences such as unemployment [13], 14]. Furthermore, the utilization of ART to treat HIV counterproductively induces sensory neuropathy through neurotoxic side effects [15], [16], [17]. Indeed, HIV-SN has an estimated 30–60 % prevalence in HIV infected patients, varying by countries and region [18]. For example, some estimates of HIV-SN are as low as 8.6 % [16] in the United States and as high as 42 % [19] in Australia. High prevalence of disease is reported in African countries with rates reaching as high as 36 % [20]. The cause of discrepancy between countries is multifactorial as delays in diagnosis, access to newer antiviral medications, and opportunistic infection rates differ by region [20]. In the post ART era, prevalence increases with advanced age, female gender, and advanced stage of HIV infection, with ART treatment itself aggravating existing neuropathy [20], [21], [22], [23], [24].

## Abnormal nociception

Physiological somatosensation begins in the skin with specialized receptors that detect touch, temperature, proprioception, and chemicals [25], 26]. Mechanoreceptors and free nerve endings transmit nociceptive signals through dorsal root ganglion (DRG) neurons to the spinal dorsal horn (SDH). Secondary afferent neurons enter the central nervous system (CNS) and ascend to higher centers like the thalamus and cerebellum [26]. Dysfunction along this pathway can lead to neuropathic pain, defined as pain from lesions or disease of the somatosensory system. Peripheral neuropathy involves distal-to-proximal “dying-back” axonal degeneration, triggered by chemical insults, trauma, or disrupted axonal transport, often involving calcium influx and axon-destructive processes [27]. Incomplete nerve repair can cause peripheral sensitization, presenting as spontaneous pain, allodynia, and hyperalgesia, with reduced intraepidermal nerve fiber density correlating with pain severity [27]. Persistent peripheral input may lead to central sensitization, where CNS neurons become hyper-responsive, manifesting as wind-up, secondary hyperalgesia, and chronic pain. Central neuropathic pain is associated with conditions such as stroke, Parkinson’s disease, spinal cord injury, and multiple sclerosis [28].

## HIV-associated sensory neuropathy (HIV-SN)

HIV cannot directly infect neurons due to lack of CD4 receptor[29]. However, many mechanisms exist by which HIV influences neuronal health. The typical life cycle of HIV begins with virion entry into CD4+ cells, which includes T cells and monocytes. The binding of gp120 to CD4+ receptors facilitate gp120 further binding to coreceptor CCR5 and CXCR4. This process mediates HIV viral entry into the cell. The initial infection phase involves integration of viral cDNA into host DNA through integrase activity [30]. Infected cells harbor integrated viral DNA and may be transcriptionally silent forming the latent viral reservoir. The post integration phase includes viral protein production, release of an immature virion and subsequent maturation, as well as secretion of viral proteins even in the absence of virus [30]. Immune cells make an ideal sanctuary for HIV, as they are widespread amongst tissues, have prolonged lifespans, and have diverse interactions with other cell types. Monocytes and macrophages exert influence on peripheral nerves and cause neuroinflammation as described below. Additionally, HIV infected monocytes (CD14+ and CD16+) transmigrate across the blood-brain barrier (BBB), resulting in CNS invasion and neuronal compromise. This activity represents a major issue in targeting HIV infection, as the CNS acts as a hidden reservoir. Even in full suppression of HIV peripherally, transcription continues to occur in the CNS, and viral reservoirs are found within the brain. The viral reservoir is protected not only from peripherally circulating pharmaceutical agents, including ART, but also from immunorecognition and elimination due to the brain’s immune privilege. This persistent, low-level expression of viral proteins contributes to ongoing neuronal dysfunction and damage, particularly in the context of chronic neuroinflammation. We will first discuss the direct mechanisms by which HIV proteins, including Tat, Viral protein R (Vpr) and gp120, cause neuronal damage. Then, we will discuss the major role of neuroinflammation as mediated by glial cell types to induce synaptic degeneration and subsequent neuropathy.

As indicated, HIV-SN occurs even in individuals with long-term controlled viremia. Most of the neuronal toxicity has been associated with the secretion of viral proteins from viral reservoirs into neighboring uninfected cells such as neurons [12], [31], [32], [33]. In agreement, exposure to the viral protein Tat for more than 48 h induced DRG apoptosis in rat models [31]. Tat can activate N-methyl-D-aspartate receptors (NMDAR) that subsequently signal for neuron apoptosis through Ca^2+^ influx [34]. Disruption of Ca^2+^ homeostasis has toxic effects on mitochondria which results in chronic energy deficits leading to abnormal electrical discharges and neuronal degeneration [35], 36]. This association with neuronal hyperexcitability is an important link to the peripheral sensitization, which contributes to neuropathy. HIV Vpr similarly induces mitotoxic effects on human DRG. Vpr induces a transient increase of Ca^2+^ in neurons leading to hyperexcitability and ultimately neuronal toxicity [9]. Additionally, Vpr is associated with neuroinflammation, as indicated by higher levels of TNF-α [9], 11]. Although the neurotoxic effects of Tat and Vpr are known, the overall contribution in HIV-SN remains uncertain. Much of the evidence implicating Tat and Vpr derives from high-concentration exposure studies that may not reflect physiologic levels in chronically infected patients [9], 31]. Furthermore, postmortem analyses have not consistently demonstrated higher levels of Tat and Vpr expression in the SDH of HIV-PAIN^+^ patients compared with HIV-PAIN^−37^. This discrepancy suggests that the relevance of Tat and Vpr *in vivo* may depend upon other factors. Overall, the current body of evidence favors gp120 as a primary driver of HIV-SN, whereas the contributions of Tat and Vpr require further investigation for clarification.

Gp120 has been implicated as a major inducer of HIV-SN [32], 37]. Gp120 expression is significantly higher in the SDH spinal cord, a pain processing center where peripheral nociceptive signals integrate into the CNS. Specifically, gp120 levels in HIV pain-positive patients were 10 times higher in the SDH than those in pain-negative patients [37]. Additionally, markers of synaptic degeneration are elevated in HIV patients with pain [12], 37], and this result is reproducible in gp120 pain mouse models via intrathecal injections (i.t.) of gp120 [37].

## Epidermal protein gene product 9.5 (PGP9.5) innervation and HIV-SN

Healthy epidermis of skin innervated with nociceptors (sensory neuronal terminals) functions to detect external stimulation such as temperature, chemical, touch, vibration and mechanical stimulation. These nociceptors can be immunopositively labeled by protein gene product 9.5 (PGP9.5^+^) in the dermis and epidermis [37], 38]. PGP 9.5^+^ was first recognized as a brain-specific protein decades ago, richly expressed in different neurons in vertebrates and forming an estimated 5–10 % of the cytoplasmic protein [39]. Within the epidermis area, only unmyelinated nociceptors (C-fiber) detect nociceptive signals and transduce nociception [40]. Pan et al. and Karlsson et al. reported that degeneration of intraepidermal nerve fiber density (IENF) occurred in both distant extremities, more obvious in distant leg, and that degeneration preceded the elevation of thermal thresholds (decreased sensitivity to heat) in neuropathy patients [41], 42]. PGP9.5^+^ is commonly used to label peripheral nerve fibers and the degeneration of the PGP9.5^+^ cutaneous nociceptor is a representative pathological biomarker of various sensory neuropathies, including HIV-SN [43], 44]. HIV-SN patients and HIV-PAIN rodent models usually associate persisting, chronic pain with progressing degeneration or even denervation of skin native PGP9.5^+^ nociceptors. According to Zhou and colleagues, IENF at the distant leg site correlates with neuropathy severity as gauged by total Neuropathy Score, a reflection of the severity of distal symmetric polyneuropathy [45]. Shi et al. reported that sural nerve autopsy showed 5/5 patients with intense ART treatment had pathological diagnosed neuropathy [46]. Additionally, Ebenezer et al. reported that the sural nerve biopsy of HIV-SN patients showed nerve fiber loss predominantly in distal terminal, including both the epidermis (IENF) and dermis, with the IENF loss being prior to loss of larger caliber and deeper dermal axons [47]. Shikuma et al. found that IENF decreases in the distal leg of HIV negative patients (30.1^#^/mm) compared to IENF in HIV positive patients with NRTI treatment (21.1^#^/mm) with further decline accompanying treatment with NRTIs [48]. These works suggest the IENF in distant limbs are the initial site of degeneration by HIV infection with ART treatment and are generally recognized as a key pathological biomarker of HIV-SN. A critical challenge is that ART cannot directly prevent IENF reduction. Previously, Mangus et al. investigated the pathogenesis of HIV-SN using simian immunodeficiency virus (SIV)-infected Asian macaques. At 84 days post viral infection, the length of PGP9.5+ nerve fiber were diminished, as measured through footpad skin biopsies [49]. This work indicates that both HIV and SIV can induce similar SN. Human samples prove the reality of HIV-SN, whereas SIV-SN was used as a close mimic of human HIV-SN. However, both human HIV and macaque SN provides limited opportunities for mechanistic analysis. For deep HIV-SN pathogenesis investigation, rodents, especially the mouse HIV-SN model, are more convenient for detailed mechanism research. Yuan et al. replicated HIV-PAIN and SN in mice by i.t. gp120 and observed that PGP9.5^+^ IENF in hind paw glabrous skin exhibited temporal degeneration post gp120 i.t, thus the mice were named as mHIV-SN mouse model. At three weeks, the innervation of plantar eccrine glands showed simultaneously the same patten of degeneration as hind paw skin, suggesting that mice developed autonomic neuropathy as well. Corresponding with the development of allodynia and PGP9.5^+^ pathologies, the envelope glycoprotein of HIV, gp120, plays an essential role in targeting SDH and sensory neurons both in humans and mice. The resulting degeneration of PGP9.5^+^ nociceptors is associated with both mouse and human HIV-PAIN and HIV-SN [37].

## HIV-SN mouse model

Yuan et al. developed mHIV-SN by i.t. of HIV-1 gp120Bal and gp120IIIB, two coreceptor tropism of gp120. I.t. injection makes gp120 easily diffuse to lumbar enlargement, an epidural area where L4, 5, 6 DRGs and spinal nerves are located and create a scenario that mimics the gp120 increase in the SDH, DRG, and lumbar spinal nerve of hHIV-SN [50]. This is the first mouse model parallel of gp120-induced nociceptive pathologies in mice and patients [37], 51]. This i.t. gp120 mHIV-SN phenocopies the behavioral, neurological, glial, synaptic, and molecular pathologies observed in human HIV-SN (hHIV-SN). Pain is a major comorbidity of hHIV-SN and has been clearly observed in mHIV-SN. Of particular importance, the mHIV-SN model develops robust ‘dying-back’ SN, manifested as denervation of PGP9.5^+^ C-fiber nerve endings in the epidermis and autonomic neuropathy, evidenced by neuroinflammation and denervation of the sweat gland. This is clinically aligned with observations that hHIV-SN is often accompanied by autonomic neuropathy [37]. Additionally, the SDH tissue from mHIV-SN and hHIV-SN show extensive pathological similarities, suggesting the relevance of mHIV-SN to hHIV-SN [37]. The strength of the mHIV-SN model to date is that pain pathologies have been confirmed to systematically phenocopy pathologies of hHIV-SN and HIV-PAIN patients. Acharjee et al. investigated the mechanisms of a cytotoxic HIV-1 accessory protein, Vpr, on human DRG neurons. Vpr caused DRG neuronal damage, rising neuronal calcium level and cytokine perturbation, suggesting its contribution to HIV-SN and HIV-PAIN [9], [52], [53], [54]. Keswani and colleagues, using didanosine on gp120 transgenic mice that expressed gp120 under a Glial Fibrillary Acidic Protein promoter, found that such mice developed distal degeneration of unmyelinated sensory axons, and C-fiber loss as seen in patients with HIV-SN [52]. Wallace et al. revealed that rats with perineural HIV-gp120 treated with zalcitabine (i.p. 50 mg/kg) showed degeneration of C fibers in the epidermis and allodynia [53]. Together, these studies suggest that both HIV-SN and NRTIs induce SN caused C-denervation, which mainly manifests as HIV-PAIN [53].

## Pathogenic role of HIV viral protein

The gp120 glycoprotein on the HIV-1 envelope mediates virus entry by binding to a CD4 receptor. Despite effective ART, neurological abnormalities persist in the post-ART era [24]. Our HIV postmortem data indicate that the development of HIV-SN associated pain was intimately linked with extraordinary high levels of gp120 protein in SDH in hHIV-SN patients (3/5) with pain even on intensified ART, compared to relatively lower levels of gp120 in hHIV-SN patients (2/5) with pain but not on ART. Additionally, levels of both the Tat and Vpr proteins were much lower than gp120 in all hHIV-SN patients with pain [37], indicating that ART can effectively the decrease viral load and improve CD4 cell counts, but cannot stop the persistent gp120 expression in SDH. This observation shows the key etiological role of gp120 (rather than viral load *per se*) in hHIV-SN, as plasma viral loads and CD4 cell counts are not closely associated with hHIV-SN [37], 55]. Moreover, gp120 transgenic mice provide *in vivo* evidence that gp120 is responsible for HIV associated nervous system impairment [56], including neuropathic pain beginning at 4 months [12]. Overall, gp120 is the critical viral protein for HIV to induce HIV-SN, a critical nociceptive mechanism for HIV-PAIN both in mice and humans.

**Figure 1:**
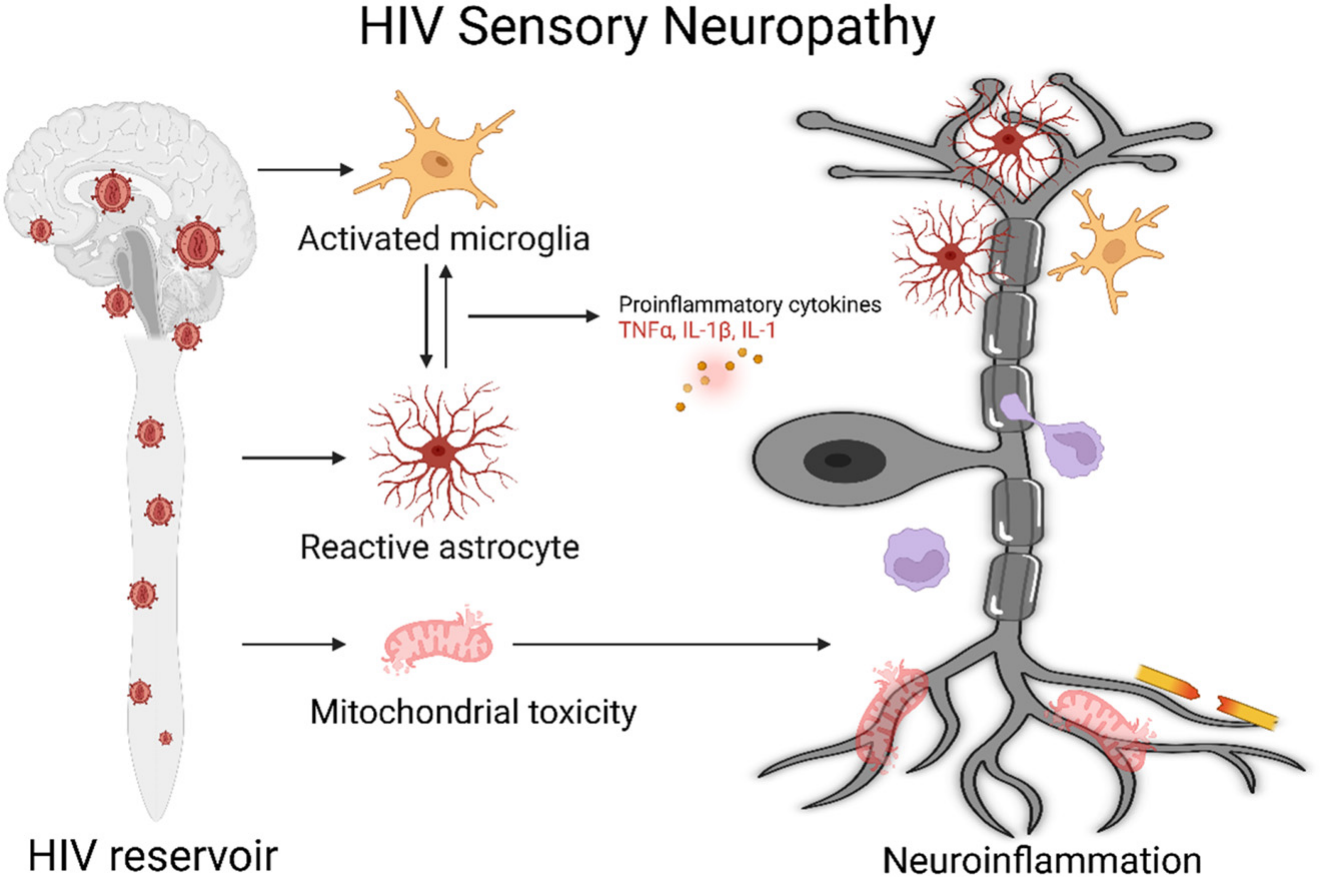
HIV reservoirs induce HIV associated sensory neuropathy through mechanisms of neuroinflammation. HIV viral proteins such as gp120, Tat and Vpr contribute to neuroinflammation via activation of astrocytes and microglia. Neurons, astrocytes, and microglia have crosstalk via expression of inflammatory cytokines. HIV viral proteins are also toxic to mitochondria of neurons. Illustration created in https://BioRender.com.

**Figure 2:**
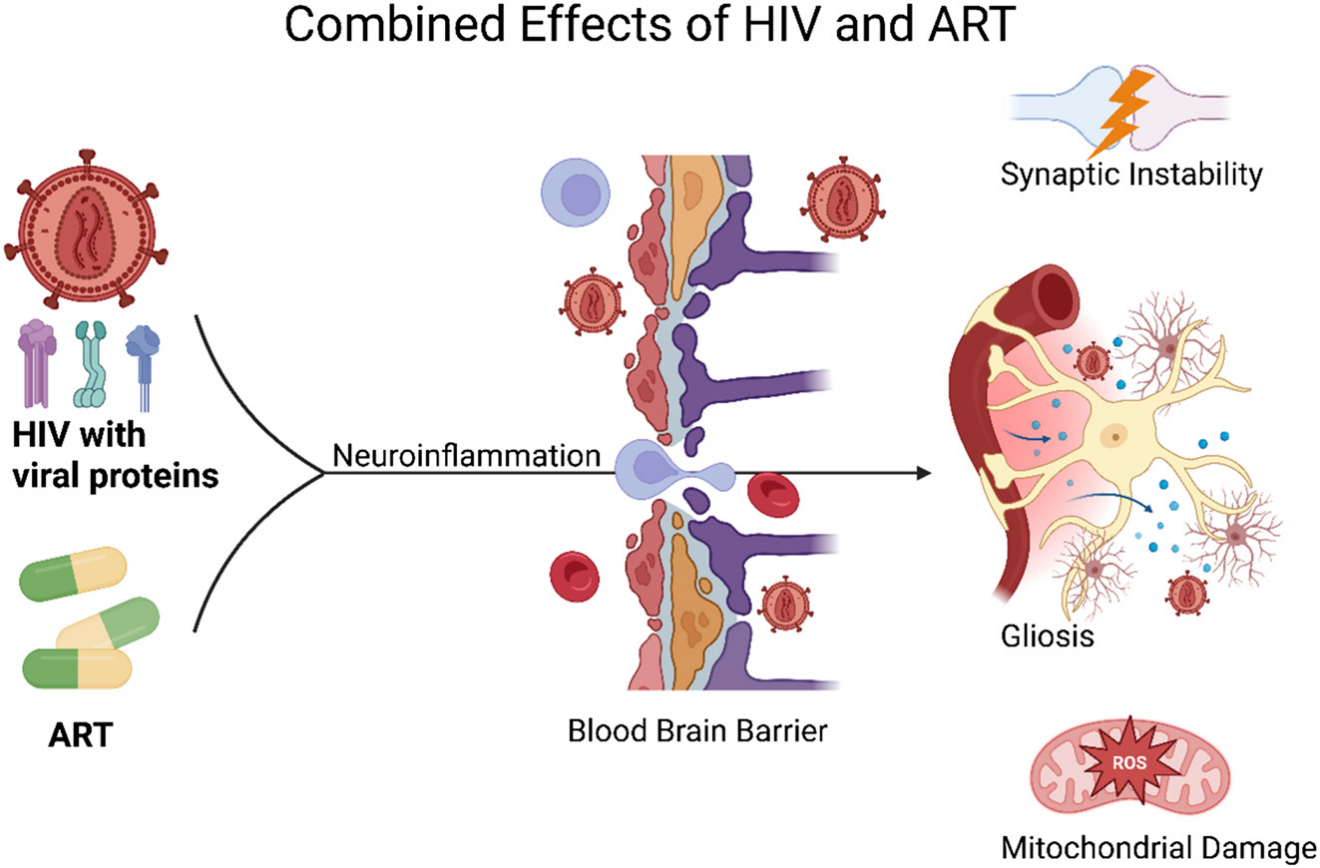
Combined effects of HIV and antiretroviral therapy (ART). The effects of HIV, including viral proteins, and ART combine to generate neuroinflammation through the Wnt5a pathway. Additionally, neuroinflammation disrupts the integrity of blood brain barrier (BBB) and permits the entry of immune cells, and HIV into the tightly regulated central nervous system (CNS). Viral protein and ART drug-induced neuroinflammation cause synaptic instability, gliosis, and damage to mitochondria. Illustration created in https://BioRender.com.

Soluble gp120 similarly exerts effects on neurons through NMDAR binding [12], 33]. Gp120 directly binds to NMDAR, triggering a signaling cascade that upregulates fractalkine and its receptor CX3CR1, leading to activation of microglia [12]. It has been further elicited that gp120 induced fractalkine upregulation is mediated by the Wnt3a/beta-catenin signaling pathway [12]. Activated microglia then contribute to synaptic degeneration, as shown by synaptic protein loss *in vitro* and *in vivo* [12]. Activated microglial cells secrete inflammatory products such as IL-1β, TNF-α, and brain-derived neurotropic factors. While microglial activation plays a significant role in the early phases of gp120-induced neuropathic pain, it is less involved in late phases [57], 58]. This is evidenced by similar levels of microglial markers, such as ionized Ca^2+^ binding adaptor molecule 1 and CD11b, in the SDH of HIV negative and HIV positive groups when evaluating chronic pain [57]. The transient nature of microglial activation is observed in rodent models post-nerve injury, with microglial proliferation beginning to decline after 7 days [59]. This result suggests that microglia do most of their damage in the acute phase of inflammation, but not chronically. This is indeed evident with microglial ablation only partially inhibiting the early phase of gp120 induced hyperalgesia without impacting the late stage of pain development in mouse models [58]. (Table 1, Figures 1 and 2).

**Table 1:** Summary of viral and ART mechanisms contributing to HIV- SN.

Agent	Mechanism	Clinical impact	Potential therapeutic intervention
Tat	Activates NMDAR → ↑Ca^2+^ influx → mitochondrial dysfunction and neuron apoptosis.	Neuronal hyperexcitability, peripheral sensitization, and neuropathic pain.	NMDA receptor antagonists and mitochondrial protectants.
Vpr	Increases neuronal Ca^2+^→ mitochondrial damage; triggers TNF-α–mediated inflammation.	Neuronal hyperexcitability, and inflammation.	Calcium channel modulators and anti-inflammatory agents.
gp120*	Binds receptors/coreceptors CD4, CXCR4, CCR5 in glia cell →activation of microglial & astrocyte cells→ neuroinflammation. Upregulates Wnt5a and enhancement of NMDAR in neuron→ neurotoxicity→ neuronal injury, central sensitization and neuroinflammation.	Allodynia in gp120 rodent pain model, chronic HIV-PAIN^+^/SN and other neurological disorders.	CCR1 antagonists, microglial inhibitors, NMDA blockers, and Wnt5a antagonists.
ART (NRTIs)	Mitochondrial toxicity and dysfunction → upregulation of Wnt5a → astrocyte activation and neuroinflammation → axonal degeneration.	Neurotoxicity, and induced peripheral neuropathy.	Replace with less neurotoxic agents, mitochondrial protectants, and Wnt5a antagonists.
CNS Viral reservoir	Latent infection in macrophages and microglia → continued secretion of viral proteins despite ART.	Chronic neuronal injury, and chronic neuroinflammation.	Latency-reversing agents, BBB-penetrant ARTs, and use of nanoparticle vehicles.

Gp120* is a primary inducer of sensory neuropathy.

The non-canonical pathway utilizing the Wnt family member 5a (Wnt5a) has been shown to be a major signal molecule in gp120 HIV-SN [60]. In this pathway, gp120 upregulates Wnt5a in neurons [58]. The release of Wnt5a activates astrocytes utilizing the receptor tyrosine kinase-like orphan receptor 2, among other receptors [58]. Now that astrogliosis is achieved, pathways including c-Jun N-terminal kinase and calcium/calmodulin-dependent protein kinase II are used to stimulate neuroinflammatory molecules [8], 37], 60]. Wnt5a expression specifically induces IL-1β, IL-1, and TNF-α, leading to neuroinflammation [8], 10]. This overexpression of inflammatory markers recruits circulating monocytes to the sites of damage and induces apoptosis, causing destruction to nearby neurons [61]. Gp120 additionally induces neural circuit polarization, whereby increased inputs to excitatory neurons and reduced excitation in inhibitory neurons cause central sensitization. This central sensitization is facilitated through astrogliosis and IL-1β and is associated with chronic pain [58].

Other cell types, including DRG resident macrophages, oligodendrocytes, and Schwann cells, have also been implicated in gp120 induced neuroinflammation, but the literature is limited on their roles in the pathogenesis of HIV-SN [12], [62], [63], [64].

## Antiretroviral therapy (ART) induced neuropathy

Nucleoside reverse transcriptase inhibitors (NRTIs) compete with natural deoxynucleotides for incorporation into a growing viral DNA chain. However, NRTIs lack a 3′-hydroxyl group on the deoxyribose moiety. This difference results in incorporating an NRTI, and the next incoming deoxynucleotide cannot form the following 5′, 3′ phosphodiester bond needed to extend the DNA chain. The result is a chain termination in DNA synthesis [65]. The usage of dNTP is a common feature of all DNA polymerases, and thus NRTIs expected to block human as well as the viral DNA polymerases and lead to toxicity [66], [67], [68]. ART, the only FDA-approved treatment for HIV infection, is composed of a cocktail of medicines targeting different stages of the viral cell cycle. A typical HIV regimen of ART generally include the “backbone” of two NRTIs and one additional class of ART [65]. Other ART classes include an integrase inhibitor, a non-nucleoside reverse transcriptase inhibitor (NNRTI) or a protease inhibitor. ART does not cure HIV, but dramatically increase life expectancy of HIV patients and is crucial for lowering the risk of transmission [2]. Rapid initiation of ART is associated with better health outcomes in HIV patients [69]. While ART therapy is a mainstay in treatment of HIV, usage of certain ART is still limited due to ART related toxicity.

ART dramatically extends the life span, decreases comorbidities and improves the quality of life of PLWH; however, life-long administration of certain ART causes neurological complications, including SN. NRTIs are especially relevant in this regard, because their neurotoxicity is well observed both in patients [70], replicated NRTI administration induced neuroinflammation in the mouse brain [71], and in the spinal cord [72]. According to the US Centers for Disease Control and Prevention, in a cohort of 2,515 PLWH on ART treatment, 329 (13.1 %) were diagnosed with HIV-SN [73]. However, the incidence may be sharply underreported. In our study, 5/5 postmortem HIV PAIN patients were pathologically diagnosed with HIV-SN by sural nerve autopsy [46]. The relative contributions of unresolved inflammation, ongoing HIV replication, and ART-related neurotoxicity may all contribute, and clinical evidence cannot definitively identify either viral infection or ART toxicity as the primary driver of HIV-SN.

Viral proteins have been shown to preferentially damage large Aβ-fiber myelinated primary afferent axons that convey touch and proprioceptive information, whereas NRTIs preferentially damage small myelinated Aδ fibers and nonmyelinated C fibers that convey temperature and pain information [43]. Peripheral neuropathy is a painful and debilitating complication associated with NRTI therapy. The dideoxy-NRTIs zalcitabine and didanosine, as well as the thymidine-analog NRTI stavudine may hinder adherence to the life-saving treatment [74], 75]. Early NRTIs, including zalcitabine, now are discontinued for clinical use. NRTIs are well-documented to induce mitochondrial dysfunction and oxidative stress. Currently HIV treatment regimens, such as Biktarvy (bictegravir/emtricitabine/tenofovir alafenamide) contain an integrase strand transfer inhibitor and two backbone medicine of NRTIs, tenofovir, emtricitabine. While current ART regimens have lower levels of toxicity than older NRTI drugs, they are still reported to induce neuropathy in clinic. Tenofovir causes peripheral neuropathy in patients [73] as well as in wild type and gp120 transgenic mice, with alterations in inflammatory signaling and mitochondrial activity in neurons [76]. Tenofovir and emtricitabine also showed neurotoxicity, with the concentration related to the drug’s reported CSF concentration level [77]. Emtricitabine is part of several recommended first-line ART, one-pill a day regiments. Emtricitabine combines with other classes of ART drugs such as Biktarvy (bictegravir/emtricitabine/tenofovir alafenamide), Atripla (efavirenz/emtricitabine/tenofovir disoproxil fumarate), Delstrigo (doravirine/lamivudine/tenofovir disoproxil fumarate) [78]. Emtricitabine is found to induce peripheral neuropathy, especially when used in combination with tenofovir. However, Margolis assessed that both emtricitabine and tenofovir are less common to cause mitochondrial toxicity compared with older NRTIs [70]. The transition to newer generation ART agents with more favorable safety profiles has also extended to HIV pre-exposure prophylaxis (PrEP) regimens such as Truvada (emtricitabine/tenofovir disoproxil fumarate), Descovy (emtricitabine/tenofovir alafenamide), and Apretude (cabotegravir). Although limited evidence suggests that PrEP usage may be associated with modest increases in immune activation [79], comprehensive safety reviews report no clinically meaningful risk of neuropathy in PrEP users [80]. This is further supported by the rare prevalence of neuropathic symptoms in large PrEP cohorts [81]. However, our preliminary data of Biktarvy (emtricitabine, tenofovir alafenamide, bictegravir) showed induction of chronic pain and neuropathy in mice (unpublished data). Currently, HIV-SN is still with significant prevalence in an era where HIV viremia is dramatically suppressed. We cannot exclude the possibility that the newer generation of ART is free from neurotoxicity and ART associated neuropathy, as the NRTIs, such as emtricitabine and tenofovir alafenamide, are still the major components of most ART polypills. Therefore, the underlying unknown pathogenesis of ART in addition to mitochondrial toxicity needs to be explored further. ART neurotoxicity studies are limited and have a long way to go.

Mitochondrial dysfunction and neuroinflammation are major mechanisms by which ART promotes neuropathy. DNA polymerase α, δ, and ε are insensitive to inhibition by dideoxynucleotides, but both DNA polymerases β and γ can be inhibited *in vitro* by these compounds [68], 74], 75], [82], [83], [84], [85], [86], [87]. As DNA polymerase γ is the only DNA polymerase involved in mitochondrial DNA (mtDNA) replication, the impact of NRTIs on this enzyme can adversely affect mitochondrial replication and function, leading to dysfunction [66], 82], [88], [89], [90]]. Studies in cell lines [91], 92], animal models [93], [94], [95], and in humans [96], [97], [98] have shown morphologic, quantitative, and functional changes of mitochondria following NRTI exposure. The mitochondrial dysfunction mechanism has been investigated for many years, with the “DNA poly-gamma hypothesis” the most prominent. This hypothesis posits that NRTIs adversely affect mitochondrial replication through binding to enzyme DNA polymerase γ and terminating DNA chain formation [17], 99]. Blockage of this protein leads to mtDNA depletion, disruption of ATP production, and increased production of free reactive oxygen species [100]. While the details of this cascade have not been fully characterized, the report of a novel, functional mtDNA POLG mutation being associated with NRTI-associated lactic acidosis [101] lends weight to the POLG inhibition hypothesis [102]. This is clinically correlated with the significant depletion of mtDNA and abnormal levels of electron transport chain proteins found in the DRG of HIV pain positive patients as opposed to HIV patients without neuropathic pain [103]. Furthermore, mitochondria represent a major metabolic hub with important connections to neuroinflammation (see review by Wang et al. [104]). Disruption in mitochondrial metabolism leads to metabolites that strongly stimulate inflammation and activate macrophages [104]. Changes to the mitochondrial membrane potential permit reactive oxygen species to accumulate leading to induction of IL-1β and mitochondria autophagy [104]. Theoretically, mitochondrial damage affects all organs and components over time (except adult red blood cells), but the inherent heterogeneity in drug absorption and mitochondrial activity dynamics make tissues with the highest energy demand most susceptible. As the nervous system is one of the most energy consuming, it is thus severely affected [105], [106], [107]. DRGs are particularly vulnerable due to their limited regenerative capacity and high metabolic demand. The defect in replication machinery ultimately impacts the oxidative phosphorylation system leading to complications that may include neurological manifestations such as peripheral neuropathy, encephalopathy, dementia, seizures, and stroke [96].

Besides mitochondrial dysfunction, other mechanisms also contribute to ART induced chronic pain and SN. Yuan et al. reported that NRTI (specifically, zalcitabine, 3′-azido-3′-deoxythymidine [AZT], lamivudine, and stavudine) administration for two weeks induced mice allodynia, with zalcitabine upregulating Wnt5a expression (SDH). Additionally observed was activation of astrocyte and microglia in the spinal cord dorsal horn of 15.5-month-old mice, which approximates 50 years in humans [108]. The mouse study aligns with that of Lichtenstein et al., who found that, in PLWH treated with NRTI regiment, being >40 years was a significant risk factor for HIV-SN. This work indicates that NRTI elicited Wnt5a mediated neuroinflammation, and inhibition of Wnt5a with Box5 blocked zalcitabine induced up-regulation of TNF-alpha, IL-1beta, and IL-6. Thus, spinal cord neuroinflammation critically contributes to zalcitabine induced HIV-SN [30]. Using a creative *drosophila* larvae model to investigate NRTI-induced SN, Bush et al. modeled AZT induced allodynia and thermal hyperalgesia in drosophila larvae fed with AZT. They found that AZT exposure increases the dynamics and instability of sensory neurons measured through terminal dendrite sprouting, growing, and retracting on live imaging. Restoring dendritic stability through Par-1 knockdown suppresses the fragmentation-like phenotype induced by AZT in flies [109]. This work built an excellent model in understanding the mechanisms of ART induced SN and provides insight on the mechanism of NRTI disrupting stability of sensory neruons [109]. Synapse stability is another factor by which ART can influence neuronal health even before the death of a neuron. As seen in other neurodegenerative diseases such as dementia, the lack of synaptic stability of dendrites indicates neuronal dysfunction [108]. Indeed, improving synapse stability in the context of NRTI induced neuropathy led to a suppression of nociception hypersensitivity in a drosophila model [109]. Synapse dysfunction is measured by synapse turnover rates, post-synaptic plasticity, and impaired circuit function [108]. AZT, an NRTI, causes synaptic instability and fragmentation of sensory dendrites [110]. Synaptic instability and dynamic dendrites are other mechanisms by which NRTI causes neurotoxicity and subsequent neuropathy.

## Combined toxicity of HIV and ART

We now explore the synergistic effects of HIV and ART in initiating neuropathy. How ART induces neuropathy is difficult to ascertain, as it may unmask subclinical HIV neuropathy or indeed add directly to neurotoxicity. In any case, ART administration has been shown to be a risk factor for developing neuropathy and those with mild neuropathy may have progressive disease in a dose-dependent manner [16], 111]. In patients with mild peripheral neuropathy, usage of NRTIs, including zalcitabine, didanosine, stavudine, and lamivudine, exacerbated symptoms [112]. The need for therapeutic strategies that minimize these adverse effects while effectively managing HIV infection is critical. So far, the mainstay in symptomatic treatment of neuropathic pain due to ART is dose adjustment or switching medication regimens. While planning a medication regiment to avoid the most neurotoxic medications is feasible in the United States, patients in poorer countries must still utilize older ARTs due to cost.

HIV-SN, a major pathology of HIV-PAIN, can be aggravated by NRTIs which are the backbone of ART. There are two critical algogens, gp120 and NRTIs which are tightly intermingled in HIV-PAIN and are impossible to untangle. Yuan et al. identified gp120 as a major algogenic protein in HIV-PAIN by comparing the HIV-PAIN mortem SDH and mouse spinal cord [37]. Shi Y et al. found that gp120 up-regulated Wnt5a expression in the mortem spinal cord of HIV-PAIN patient and the mouse spinal cord [54], 113]. Wnt5a is a neuronal secreted protein which is physiologically necessary for neuron excitation and pathologically sensitizes sensory neurons [54]. Li B et al. reported how gp120 through Wnt5a regulated the downstream inflammatory cytokines IL-1β, IL-1, and TNF-α secretion [8]. Yuan et al. observed that Wnt5a antagonism through Box-5 attenuated gp120-induced mechanical allodynia. Conversely, a Wnt5a agonist such as Foxy5 facilitated the allodynia. A JNK/TNF- α pathway is the downstream effector of Wnt5a, and a JNK-specific inhibitor SP600125 blocked either gp120- or Foxy5-induced allodynia as evidenced through Von Frey testing and SDH neuronal spiking evoked via mechanical stimulation of the hind paw [60]. Yuan et al. also reported that NRTIs upregulate the same Wnt5a pathway, leading to astrogliosis and the inflammatory response [71], 72]. The result showed that HIV and NRTIs form a double assault on the pain circuit through Wnt5a mediated astrogliosis, SDH sensitization and neuroinflammation. HIV-SN, signaled as cutaneous PGP9.5^+^ denervation, is a pathological mechanism for HIV-PAIN [37]. Similarly, Gp120 or Wnt5a agonism both cause PGP9.5^+^ denervation (unpublished data). Wnt5a antagonism has attenuated both gp120 and NRTI induced pain. A potential safety strategy for HIV-PAIN and HIV-SN that specifically modulates the Wnt5a pathway might be a solution. Wnt5a upregulation is found both in gp120 pain mouse model and mortem SDH, as well as in the NRTI induced process leading to chronic pain and neuroinflammation [8], 60], 113]. Further research can identify whether these combined effects constitute a true multiplicative effect. Notably, treatment of HIV-PAIN with opioids can temporally alleviate severe pain, but repeated morphine is another trigger in upregulating the Wnt5a pathway and worsens the existing allodynia and promoted astrogliosis in the SDH in mice [114]. The activation of the Wnt5a pathway leads to upregulation of pro-inflammatory cytokines IL-1β and TNF-α in mice and heightens the existing HIV-PAIN [58], 72], 114]. Thus, opioids are not a good option to treat severe neuropathic pain for PLWH. Once again, astrogliosis through the Wnt5a pathway is the key step in the pathogenesis of neuropathic pain and targeting Wnt5a might be a promising approach as a non-opioid analgesic.

Entering the CNS is a critical step in HIV-SN and early entry of HIV to the brain (1 week) facilitates the generation of viral reservoir. The primary way for HIV to initially enter the CNS is through infected monocytes by increasing monocyte chemoattractant protein-1 (CCL2) and viral replication dependent mechanisms [115]. The transmigration of infected monocytes triggers BBB disruption and short/long term neuronal and glial compromise. Disruption of this regulatory barrier allows harmful substances from the peripheral circulation to enter the CNS, thereby promoting viral entry and increasing the vulnerability to inflammation driven by immune cell activity. HIV and ART share a mechanism that facilitates increased HIV penetration into the CNS [116]. Gp120 is linked to increased permeability of the BBB *in vitro* and *in vivo* [116]. The primary mechanism of BBB disruption involves activation of neuroinflammation and increase of cytokines such as TNF-α and CCL2 [117]. Furthermore, ARTs, including Efavirenz (an NNRTI) and Dolutegravir (integrase inhibitor), cause increased BBB permeability through the disruption of tight junction structures and increased inflammatory response [32]. Thus, interference with the BBB facilitates a process by which a positive feedback loop of pathogenic molecules may enter the CNS. While this process does have some positive effect, by allowing ART to be effective in reducing the HIV viral load in the normally hidden reservoir of the CNS, at the same time, the protective function of the BBB is also diminished. Once again, a paradoxical effect of HIV and ART is observed through interactions at the BBB, further compromising neuronal/glial function and intensifying HIV-SN.

## Conclusions

Despite the significant progress in managing HIV with ART, complications such as HIV-SN remain a critical concern. The interplay between HIV and ART-induced neurotoxicity exacerbates neuropathic pain through acute and chronic mechanisms, including direct viral protein toxicity, neuroinflammation, mitochondrial dysfunction, and BBB disruption. While ART has been pivotal in increasing the life expectancy of PLWH, neurotoxic effects of certain agents contribute to the burden of HIV-SN [21]. Current treatment options for HIV-SN are limited to ART modification with selection of low-risk ART and symptomatic pain management. The shared involvement of the Wnt5a signaling pathway in both HIV and ART-induced neuropathy highlights a key target for future therapeutic interventions. Furthermore, the paradoxical effects of ART on BBB integrity present challenges in balancing effective viral suppression with minimizing neurotoxicity. The use of nanomedicine as a vehicle to introduce ART to the CNS reservoir of HIV without disrupting the BBB could be effective in addressing this issue. An *in vitro* study from Nair et al. showed promising results of delivering ART via magneto-electro nanoparticles to the CNS [118]. After crossing the BBB, these nanoparticles are uniquely equipped to release their medication on demand to achieve higher therapeutic levels once in a latent reservoir. Other forms of nanoparticles including gold, metal oxides and core-shell formulations have also been shown to improve bioavailability with low overall toxicity [119], 120]. Utilizing novel clustered regulatory interspaced short palindromic repeat (CRISPR) is another promising strategy against HIV infection that will also benefit in nanoparticle delivery [121], 122]. Confronting the mechanisms underlying HIV-SN will be essential in reducing the long-term neurological burden associated with chronic HIV. A deeper understanding of these processes paves the way for targeted therapeutic interventions beyond symptomatic pain management. Future efforts should focus on treatments that address both the neurodegenerative effects of HIV and the adverse neurological impacts of ART, ultimately improving the quality of life for individuals living with HIV.

## References

[1] UNAIDS. Global HIV Statistics (2025). UNAIDS 2024 epidemiological estimates. ..

[2] Trickey A, Sabin CA, Burkholder G, Crane H, d’Arminio Monforte A, Egger M (2023). Life expectancy after 2015 of adults with HIV on long-term antiretroviral therapy in Europe and North America: a collaborative analysis of cohort studies. The Lancet HIV.

[3] Cervia LD, McGowan JP, Weseley AJ (2010). Clinical and demographic variables related to pain in HIV-infected individuals treated with effective, combination antiretroviral therapy (cART). Pain Med.

[4] Bruce RD, Merlin J, Lum PJ, Ahmed E, Alexander C, Corbett AH (2017). 2017 HIVMA of IDSA clinical practice guideline for the management of chronic pain in patients living with HIV. Clin Infect Dis.

[5] Thakur KT, Boubour A, Saylor D, Das M, Bearden DR, Birbeck GL (2019). Global HIV neurology: a comprehensive review. Aids.

[6] Gabbai AA, Castelo A, Oliveira AS (2013). HIV peripheral neuropathy. Handb Clin Neurol.

[7] Benjamin LA, Bryer A, Emsley HC, Khoo S, Solomon T, Connor MD (2012). HIV infection and stroke: current perspectives and future directions. Lancet Neurol.

[8] Li B, Shi Y, Shu J, Gao J, Wu P, Tang SJ (2013). Wingless-type mammary tumor virus integration site family, member 5A (Wnt5a) regulates human immunodeficiency virus type 1 (HIV-1) envelope glycoprotein 120 (gp120)-induced expression of pro-inflammatory cytokines via the Ca2+/calmodulin-dependent protein kinase II (CaMKII) and c-Jun N-terminal kinase (JNK) signaling pathways. J Biol Chem.

[9] Acharjee S, Noorbakhsh F, Stemkowski PL, Olechowski C, Cohen EA, Ballanyi K (2010). HIV-1 viral protein R causes peripheral nervous system injury associated with in vivo neuropathic pain. Faseb J.

[10] Shi Y, Yuan S, Tang SJ (2019). Morphine and HIV-1 gp120 cooperatively promote pathogenesis in the spinal pain neural circuit. Mol Pain.

[11] Williams ME, Williams AA, Naudé PJW (2023). Viral protein R (Vpr)-induced neuroinflammation and its potential contribution to neuronal dysfunction: a scoping review. BMC Infect Dis.

[12] Ru W, Liu X, Bae C, Shi Y, Walikonis R, Mo Chung J (2019). Microglia mediate HIV-1 gp120-Induced synaptic degeneration in spinal pain neural circuits. J Neurosci.

[13] Anastasi JK, Pakhomova AM (2020). Assessment and management of HIV distal sensory peripheral neuropathy: understanding the symptoms. J Nurse Pract.

[14] Alford K, Daley S, Banerjee S, Hamlyn E, Trotman D, Vera JH (2022). “A fog that impacts everything”: a qualitative study of health-related quality of life in people living with HIV who have cognitive impairment. Qual Life Res.

[15] Cohen BA, Bartt R, Georgiev V, Skowron G, Ogden R, Lange JMA (2006). Peripheral neuropathy associated with nucleoside reverse transcriptase inhibitor therapy. Reverse transcriptase inhibitors in HIV/AIDS therapy.

[16] Evans SR, Ellis RJ, Chen H, Yeh T, Lee AJ, Schifitto G (2011). Peripheral neuropathy in HIV: prevalence and risk factors. Aids.

[17] Hao S (2013). The molecular and pharmacological mechanisms of HIV-related neuropathic pain. Curr Neuropharmacol.

[18] Julian T, Rekatsina M, Shafique F, Zis P (2021). Human immunodeficiency virus-related peripheral neuropathy: a systematic review and meta-analysis. Eur J Neurol.

[19] Smyth K, Affandi JS, McArthur JC, Bowtell-Harris C, Mijch A, Watson K (2007). Prevalence of and risk factors for HIV-associated neuropathy in Melbourne, Australia 1993-2006. HIV Med.

[20] Tassew WC, Gebiru AM, Getnet M, Mengistie BA, Bitew DA, Getahun AB (2025). Sensory neuropathy and associated factors among patients living with human immuno-deficiency virus in Africa: a systematic review and meta-analysis. Virol J.

[21] Winias S, Radithia D, Savitri Ernawati D (2020). Neuropathy complication of antiretroviral therapy in HIV/AIDS patients. Oral Dis.

[22] Nikolaidis I, Karakasi MV, Bakirtzis C, Skoura L, Pilalas D, Boziki MK (2022). Epidemiology of HIV-associated peripheral neuropathy in people living with human immunodeficiency virus infection in Greece. Int J STD AIDS.

[23] Ngassa Mbenda HG, Wadley A, Lombard Z, Cherry C, Price P, Kamerman P (2017). Genetics of HIV-associated sensory neuropathy and related pain in Africans. J Neurovirol.

[24] Kamerman PR, Moss PJ, Weber J, Wallace VC, Rice AS, Huang W (2012). Pathogenesis of HIV-associated sensory neuropathy: evidence from in vivo and in vitro experimental models. J Peripher Nerv Syst.

[25] Julius D, Basbaum AI (2001). Molecular mechanisms of nociception. Nature.

[26] Wang L-H, Ding W-Q, Sun Y-G (2022). Spinal ascending pathways for somatosensory information processing. Trends Neurosci.

[27] Wang JT, Medress ZA, Barres BA (2012). Axon degeneration: molecular mechanisms of a self-destruction pathway. J Cell Biol.

[28] Colloca L, Ludman T, Bouhassira D, Baron R, Dickenson AH, Yarnitsky D (2017). Neuropathic pain. Nat Rev Dis Primers.

[29] Kovalevich J, Langford D (2012). Neuronal toxicity in HIV CNS disease. Future Virol.

[30] Rossi E, Meuser ME, Cunanan CJ, Cocklin S (2021). Structure, function, and interactions of the HIV-1 capsid protein. Life.

[31] Chi X, Amet T, Byrd D, Chang KH, Shah K, Hu N (2011). Direct effects of HIV-1 Tat on excitability and survival of primary dorsal root ganglion neurons: possible contribution to HIV-1-associated pain. PLoS One.

[32] Huang J, Lin F, Hu Y, Bloe CB, Wang D, Zhang W (2022). From initiation to maintenance: HIV-1 Gp120-induced neuropathic pain exhibits different molecular mechanisms in the mouse spinal cord via bioinformatics analysis based on RNA sequencing. J Neuroimmune Pharmacol.

[33] Zhou Y, Liu J, Xiong H (2017). HIV-1 glycoprotein 120 enhancement of N-Methyl-D-Aspartate NMDA receptor-mediated excitatory postsynaptic currents: implications for HIV-1-associated neural injury. J Neuroimmune Pharmacol.

[34] Kim HJ, Martemyanov KA, Thayer SA (2008). Human immunodeficiency virus protein tat induces synapse loss via a reversible process that is distinct from cell death. J Neurosci.

[35] Bennett GJ, Doyle T, Salvemini D (2014). Mitotoxicity in distal symmetrical sensory peripheral neuropathies. Nat Rev Neurol.

[36] Verma M, Lizama BN, Chu CT (2022). Excitotoxicity, calcium and mitochondria: a triad in synaptic neurodegeneration. Transl Neurodegener.

[37] Yuan SB, Shi Y, Chen J, Zhou X, Li G, Gelman BB (2014). Gp120 in the pathogenesis of human immunodeficiency virus-associated pain. Ann Neurol.

[38] Simone DA, Nolano M, Johnson T, Wendelschafer-Crabb G, Kennedy WR (1998). Intradermal injection of capsaicin in humans produces degeneration and subsequent reinnervation of epidermal nerve fibers: correlation with sensory function. J Neurosci.

[39] Day IN, Thompson RJ (2010). UCHL1 (PGP 9.5): neuronal biomarker and ubiquitin system protein. Prog Neurobiol.

[40] Olson W, Abdus-Saboor I, Cui L, Burdge J, Raabe T, Ma M (2017). Sparse genetic tracing reveals regionally specific functional organization of mammalian nociceptors. Elife.

[41] Pan CL, Lin YH, Lin WM, Tai TY, Hsieh ST (2001). Degeneration of nociceptive nerve terminals in human peripheral neuropathy. Neuroreport.

[42] Karlsson P, Porretta-Serapiglia C, Lombardi R, Jensen TS, Lauria G (2013). Dermal innervation in healthy subjects and small fiber neuropathy patients: a stereological reappraisal. J Peripher Nerv Syst.

[43] Kokotis P, Schmelz M, Papadimas GK, Skopelitis EE, Aroni K, Kordossis T (2013). Polyneuropathy induced by HIV disease and antiretroviral therapy. Clin Neurophysiol.

[44] Pardo CA, McArthur JC, Griffin JW (2001). HIV neuropathy: insights in the pathology of HIV peripheral nerve disease. J Peripher Nerv Syst.

[45] Zhou L, Kitch DW, Evans SR, Hauer P, Raman S, Ebenezer GJ (2007). Correlates of epidermal nerve fiber densities in HIV-associated distal sensory polyneuropathy. Neurology.

[46] Shi Y, Gelman BB, Lisinicchia JG, Tang SJ (2012). Chronic-pain-associated astrocytic reaction in the spinal cord dorsal horn of human immunodeficiency virus-infected patients. J Neurosci.

[47] Ebenezer GJ, McArthur JC, Thomas D, Murinson B, Hauer P, Polydefkis M (2007). Denervation of skin in neuropathies: the sequence of axonal and Schwann cell changes in skin biopsies. Brain.

[48] Shikuma CM, Bennett K, Ananworanich J, Gerschenson M, Teeratakulpisarn N, Jadwattanakul T (2015). Distal leg epidermal nerve fiber density as a surrogate marker of HIV-associated sensory neuropathy risk: risk factors and change following initial antiretroviral therapy. J Neurovirol.

[49] Mangus LM, Dorsey JL, Weinberg RL, Ebenezer GJ, Hauer P, Laast VA (2016). Tracking epidermal nerve fiber changes in Asian macaques: tools and techniques for quantitative assessment. Toxicol Pathol.

[50] Rahman MM, Lee JY, Kim YH, Park CK (2023). Epidural and intrathecal drug delivery in rats and mice for experimental research: fundamental concepts, techniques, precaution, and application. Biomedicines.

[51] Milligan ED, O’Connor KA, Nguyen KT, Armstrong CB, Twining C, Gaykema RPA (2001). Intrathecal HIV-1 envelope glycoprotein gp120 induces enhanced pain states mediated by spinal cord proinflammatory cytokines. J Neurosci.

[52] Keswani SC, Jack C, Zhou C, Hoke A (2006). Establishment of a rodent model of HIV-associated sensory neuropathy. J Neurosci.

[53] Wallace VC, Blackbeard J, Segerdahl AR, Hasnie F, Pheby T, McMahon SB (2007). Characterization of rodent models of HIV-gp120 and anti-retroviral-associated neuropathic pain. Brain.

[54] Shi Y, Yuan S, Li B, Wang J, Carlton SM, Chung K (2012). Regulation of Wnt signaling by nociceptive input in animal models. Mol Pain.

[55] Schifitto G, McDermott MP, McArthur JC, Marder K, Sacktor N, McClernon DR (2005). Markers of immune activation and viral load in HIV-associated sensory neuropathy. Neurology.

[56] Toggas SM, Masliah E, Rockenstein EM, Rall GF, Abraham CR, Mucke L (1994). Central nervous system damage produced by expression of the HIV-1 coat protein gp120 in transgenic mice. Nature.

[57] Shi Y, Gelman BB, Lisinicchia JG, Tang S-J (2012). Chronic-pain-associated astrocytic reaction in the spinal cord dorsal horn of human immunodeficiency virus-infected patients. J Neurosci.

[58] Liu X, Bae C, Gelman BB, Chung JM, Tang SJ (2022). A neuron-to-astrocyte Wnt5a signal governs astrogliosis during HIV-associated pain pathogenesis. Brain.

[59] Kohno K, Kitano J, Kohro Y, Tozaki-Saitoh H, Inoue K, Tsuda M (2018). Temporal kinetics of microgliosis in the spinal dorsal horn after peripheral nerve injury in rodents. Biol Pharm Bull.

[60] Yuan SB, Ji G, Li B, Andersson T, Neugebauer V, Tang SJ (2015). A Wnt5a signaling pathway in the pathogenesis of HIV-1 gp120-induced pain. Pain.

[61] Tian C, Ao Z, Cerneckis J, Cai H, Chen L, Niu H (2025). Understanding monocyte-driven neuroinflammation in Alzheimer’s disease using human brain organoid microphysiological systems. bioRxiv.

[62] Yu X, Liu H, Hamel KA, Morvan MG, Yu S, Leff J (2020). Dorsal root ganglion macrophages contribute to both the initiation and persistence of neuropathic pain. Nat Commun.

[63] Shi Y, Shu J, Liang Z, Yuan S, Tang S-J (2016). Oligodendrocytes in HIV-associated pain pathogenesis. Mol Pain.

[64] Keswani SC, Polley M, Pardo CA, Griffin JW, McArthur JC, Hoke A (2003). Schwann cell chemokine receptors mediate HIV-1 gp120 toxicity to sensory neurons. Ann Neurol.

[65] Kemnic TR, Gulick PG (2022). HIV antiretroviral therapy.

[66] Lewis W, Dalakas MC (1995). Mitochondrial toxicity of antiviral drugs. Nat Med.

[67] Wang TS (1991). Eukaryotic DNA polymerases. Annu Rev Biochem.

[68] Wright GE, Brown NC (1990). Deoxyribonucleotide analogs as inhibitors and substrates of DNA polymerases. Pharmacol Ther.

[69] Lundgren JD, Babiker AG, Gordin F, Emery S, Grund B, Sharma S (2015). Initiation of antiretroviral therapy in early asymptomatic HIV infection. N Engl J Med.

[70] Margolis AM, Heverling H, Pham PA, Stolbach A (2014). A review of the toxicity of HIV medications. J Med Toxicol.

[71] Wu T, Zhang J, Geng M, Tang SJ, Zhang W, Shu J (2017). Nucleoside reverse transcriptase inhibitors (NRTIs) induce proinflammatory cytokines in the CNS via Wnt5a signaling. Sci Rep.

[72] Yuan S, Shi Y, Guo K, Tang SJ (2018). Nucleoside reverse transcriptase inhibitors (NRTIs) induce pathological pain through Wnt5a-mediated neuroinflammation in aging mice. J Neuroimmune Pharmacol.

[73] Lichtenstein KA, Armon C, Baron A, Moorman AC, Wood KC, Holmberg SD (2005). Modification of the incidence of drug-associated symmetrical peripheral neuropathy by host and disease factors in the HIV outpatient study cohort. Clin Infect Dis.

[74] Yarchoan R, Mitsuya H, Broder S (1990). Immunologic issues in anti-retroviral therapy. Immunol Today.

[75] Yarchoan R, Perno CF, Thomas RV, Klecker RW, Allain JP, Wills RJ (1988). Phase I studies of 2′,3′-dideoxycytidine in severe human immunodeficiency virus infection as a single agent and alternating with zidovudine (AZT). Lancet.

[76] Fields JA, Swinton MK, Carson A, Soontornniyomkij B, Lindsay C, Han MM (2019). Tenofovir disoproxil fumarate induces peripheral neuropathy and alters inflammation and mitochondrial biogenesis in the brains of mice. Sci Rep.

[77] Robertson K, Liner J, Meeker RB (2012). Antiretroviral neurotoxicity. J Neurovirol.

[78] Julg B, Bogner JR (2008). Atriplatrade mark – HIV therapy in one pill. Ther Clin Risk Manag.

[79] Robertson J, Edén A, Nyström K, Hagberg L, Yilmaz A, Gostner JM (2021). Increased immune activation and signs of neuronal injury in HIV-negative people on preexposure prophylaxis. AIDS.

[80] Chou R, Spencer H, Bougatsos C, Blazina I, Ahmed A, Selph S (2023). Preexposure prophylaxis for the prevention of HIV: updated evidence report and systematic review for the US preventive services task force. JAMA.

[81] Owino F, Mandala J, Ambia J, Agot K, Van Damme L (2013). Neurological syndrome in an HIV-prevention trial participant randomized to daily tenofovir disoproxil fumarate (300 mg) and emtricitabine (200 mg) in Bondo, Kenya. Int Med Case Rep J.

[82] Chen CH, Cheng YC (1992). The role of cytoplasmic deoxycytidine kinase in the mitochondrial effects of the anti-human immunodeficiency virus compound, 2′,3′-dideoxycytidine. J Biol Chem.

[83] Cherrington JM, Allen SJ, McKee BH, Chen MS (1994). Kinetic analysis of the interaction between the diphosphate of (S)-1-(3-hydroxy-2-phosphonylmethoxypropyl)cytosine, ddCTP, AZTTP, and FIAUTP with human DNA polymerases beta and gamma. Biochem Pharmacol.

[84] Gray NM, Marr CL, Penn CR, Cameron JM, Bethell RC (1995). The intracellular phosphorylation of (-)-2′-deoxy-3′-thiacytidine (3TC) and the incorporation of 3TC 5′-monophosphate into DNA by HIV-1 reverse transcriptase and human DNA polymerase gamma. Biochem Pharmacol.

[85] Huang P, Farquhar D, Plunkett W (1992). Selective action of 2′,3′-didehydro-2′,3′-dideoxythymidine triphosphate on human immunodeficiency virus reverse transcriptase and human DNA polymerases. J Biol Chem.

[86] Huang P, Farquhar D, Plunkett W (1990). Selective action of 3′-azido-3′-deoxythymidine 5′-triphosphate on viral reverse transcriptases and human DNA polymerases. J Biol Chem.

[87] Parker WB, White EL, Shaddix SC, Ross LJ, Buckheit RW, Germany JM (1991). Mechanism of inhibition of human immunodeficiency virus type 1 reverse transcriptase and human DNA polymerases alpha, beta, and gamma by the 5′-triphosphates of carbovir, 3′-azido-3′-deoxythymidine, 2′,3′-dideoxyguanosine and 3′-deoxythymidine. A novel RNA template for the evaluation of antiretroviral drugs. J Biol Chem.

[88] Chen CH, Vazquez-Padua M, Cheng YC (1991). Effect of anti-human immunodeficiency virus nucleoside analogs on mitochondrial DNA and its implication for delayed toxicity. Mol Pharmacol.

[89] Cheng YC, Gao WY, Chen CH, Vazquez-Padua M, Starnes MC (1990). DNA polymerases versus HIV reverse transcriptase in AIDS therapy. Ann N Y Acad Sci.

[90] Yarchoan R, Mitsuya H, Myers CE, Broder S (1989). Clinical pharmacology of 3′-azido-2′,3′-dideoxythymidine (zidovudine) and related dideoxynucleosides. N Engl J Med.

[91] Höschele D (2006). Cell culture models for the investigation of NRTI-induced mitochondrial toxicity. Relevance for the prediction of clinical toxicity. Toxicol In Vitro.

[92] Kakuda TN (2000). Pharmacology of nucleoside and nucleotide reverse transcriptase inhibitor-induced mitochondrial toxicity. Clin Ther.

[93] Dagan T, Sable C, Bray J, Gerschenson M (2002). Mitochondrial dysfunction and antiretroviral nucleoside analog toxicities: what is the evidence?. Mitochondrion.

[94] Lewis W, Haase CP, Raidel SM, Russ RB, Sutliff RL, Hoit BD (2001). Combined antiretroviral therapy causes cardiomyopathy and elevates plasma lactate in transgenic AIDS mice. Lab Invest.

[95] Lewis W, Gonzalez B, Chomyn A, Papoian T (1992). Zidovudine induces molecular, biochemical, and ultrastructural changes in rat skeletal muscle mitochondria. J Clin Investig.

[96] Brinkman K, ter Hofstede HJ, Burger DM, Smeitink JA, Koopmans PP (1998). Adverse effects of reverse transcriptase inhibitors: mitochondrial toxicity as common pathway. Aids.

[97] Carr A, Cooper DA (2000). Adverse effects of antiretroviral therapy. Lancet.

[98] Walker UA, Bickel M, Lütke Volksbeck SI, Ketelsen UP, Schöfer H, Setzer B (2002). Evidence of nucleoside analogue reverse transcriptase inhibitor – associated genetic and structural defects of mitochondria in adipose tissue of HIV-infected patients. J Acquir Immune Defic Syndr.

[99] Hung KM, Chen PC, Hsieh HC, Calkins MJ (2017). Mitochondrial defects arise from nucleoside/nucleotide reverse transcriptase inhibitors in neurons: potential contribution to HIV-associated neurocognitive disorders. Biochim Biophys Acta Mol Basis Dis.

[100] Haynes J, Joshi A, Larue RC, Eisenmann ED, Govindarajan R (2024). Nucleoside reverse transcriptase inhibitor (NRTI)-induced neuropathy and mitochondrial toxicity: limitations of the Poly-γ hypothesis and the potential roles of autophagy and drug transport. Pharmaceutics.

[101] Yamanaka H, Gatanaga H, Kosalaraksa P, Matsuoka-Aizawa S, Takahashi T, Kimura S (2007). Novel mutation of human DNA polymerase gamma associated with mitochondrial toxicity induced by anti-HIV treatment. J Infect Dis.

[102] Lewis W (2007). Pharmacogenomics, toxicogenomics, and DNA polymerase gamma. J Infect Dis.

[103] Boustani A, Kulbe JR, Andalibi MS, Pérez-Santiago J, Mehta SR, Ellis RJ (2024). Mitochondrial DNA and electron transport chain protein levels are altered in peripheral nerve tissues from donors with HIV sensory neuropathy: a pilot study. Int J Mol Sci.

[104] Wang Y, Li N, Zhang X, Horng T (2021). Mitochondrial metabolism regulates macrophage biology. J Biol Chem.

[105] Lewis W, Griniuviene B, Tankersley KO, Levine ES, Montione R, Engelman L (1997). Depletion of mitochondrial DNA, destruction of mitochondria, and accumulation of lipid droplets result from fialuridine treatment in woodchucks (Marmota monax). Lab Invest.

[106] Wallace DC (1992). Diseases of the mitochondrial DNA. Annu Rev Biochem.

[107] Sokoloff L, Greger R, Windhorst U (1996). Cerebral metabolism and visualization of cerebral activity. Comprehensive human physiology: from cellular mechanisms to integration.

[108] Jackson JS, Witton J, Johnson JD, Ahmed Z, Ward M, Randall AD (2017). Altered synapse stability in the early stages of tauopathy. Cell Rep.

[109] Bush KM, Barber KR, Martinez JA, Tang S-J, Wairkar YP (2021). Drosophila model of anti-retroviral therapy induced peripheral neuropathy and nociceptive hypersensitivity. Biology Open.

[110] Bush KM, Barber KR, Martinez JA, Tang SJ, Wairkar YP (2021). Drosophila model of anti-retroviral therapy induced peripheral neuropathy and nociceptive hypersensitivity. Biol Open.

[111] Pahuja M, Grobler A, Glesby MJ, Karim F, Parker G, Gumede S (2012). Effects of a reduced dose of stavudine on the incidence and severity of peripheral neuropathy in HIV-infected adults in South Africa. Antivir Ther.

[112] Jones MR, Urits I, Wolf J, Corrigan D, Colburn L, Peterson E (2020). Drug-induced peripheral neuropathy: a narrative review. Curr Clin Pharmacol.

[113] Shi Y, Shu J, Gelman BB, Lisinicchia JG, Tang SJ (2013). Wnt signaling in the pathogenesis of human HIV-associated pain syndromes. J Neuroimmune Pharmacol.

[114] Shi Y, Yuan S, Tang SJ (2021). Reactive oxygen species (ROS) are critical for morphine exacerbation of HIV-1 gp120-Induced pain. J Neuroimmune Pharmacol.

[115] Osborne O, Peyravian N, Nair M, Daunert S, Toborek M (2020). The paradox of HIV blood-brain barrier penetrance and antiretroviral drug delivery deficiencies. Trends Neurosci.

[116] Strazza M, Pirrone V, Wigdahl B, Nonnemacher MR (2011). Breaking down the barrier: the effects of HIV-1 on the blood-brain barrier. Brain Res.

[117] Zhao C, Ling Z, Newman MB, Bhatia A, Carvey PM (2007). TNF-alpha knockout and minocycline treatment attenuates blood-brain barrier leakage in MPTP-treated mice. Neurobiol Dis.

[118] Nair M, Guduru R, Liang P, Hong J, Sagar V, Khizroev S (2013). Externally controlled on-demand release of anti-HIV drug using magneto-electric nanoparticles as carriers. Nat Commun.

[119] Fotooh Abadi L, Kumar P, Paknikar K, Gajbhiye V, Kulkarni S (2023). Tenofovir-tethered gold nanoparticles as a novel multifunctional long-acting anti-HIV therapy to overcome deficient drug delivery-: an in vivo proof of concept. J Nanobiotechnol.

[120] Nair M, Jayant RD, Kaushik A, Sagar V (2016). Getting into the brain: potential of nanotechnology in the management of NeuroAIDS. Adv Drug Deliv Rev.

[121] Hussein M, Molina MA, Berkhout B, Herrera-Carrillo E (2023). A CRISPR-cas cure for HIV/AIDS. Int J Mol Sci.

[122] Lee K, Conboy M, Park HM, Jiang F, Kim HJ, Dewitt MA (2017). Nanoparticle delivery of Cas9 ribonucleoprotein and donor DNA in vivo induces homology-directed DNA repair. Nat Biomed Eng.

